# 氩等离子体凝固联合分叉型被膜金属内支架置入治疗气管隆突周围复合狭窄和气管食管瘘

**DOI:** 10.3779/j.issn.1009-3419.2010.09.11

**Published:** 2010-09-20

**Authors:** 洪武 王, 凌飞 罗, 云芝 周, 洪明 马, 晶 李, 珩 邹, 冬妹 李, 楠 张

**Affiliations:** 100028 北京，煤炭总医院肿瘤微创治疗中心 Minimal Invasive Tumor Therapy Center, Meitan General Hospital, Beijing 100028, China

**Keywords:** 肿瘤, 内支架, 支气管镜, 介入放射学, Neoplasms, Stent, Bronchoscopy, Interventional radiology

## Abstract

**背景与目的:**

气管下端、隆突和双侧主支气管发生的狭窄称为多发性狭窄或复合狭窄，治疗非常棘手。本研究旨在探讨氩等离子体凝固(argon plasma coagulation, APC)联合分叉型被膜金属内支架(covered Z-type stents, CZTS)置入治疗气管隆突周围狭窄和气管食管瘘的安全性和疗效。

**方法:**

回顾性分析32例气道病变的患者，在支气管镜引导下行APC，在X线透视和/或支气管镜引导下置入CZTS。

**结果:**

19例伴有气道狭窄的患者先行APC，术前气道狭窄发生率为57.4%-72.1%，术后均明显缓解(气道狭窄发生率仅为12.8%-25.8%)。32例患者中30例技术上成功放置32个支架，2例失败。APC及成功放置支架的患者术后气促指数明显下降，KPS明显升高。13例气管下端瘘口置入分叉型CZTS，12例(92.3%)达临床治愈。术中、术后未出现大出血等并发症。两种术式术后短期内均产生大量分泌物，用气管镜可有效清除。置入内支架的患者1个月后易在支架两端形成肉芽，用APC结合冷冻可有效处理。

**结论:**

使用APC联合CZTS置入治疗气道复合狭窄和气管食管瘘快速有效，安全可靠。

## Abstract (en)

**Background and objective:**

It is a complex problem and difficult treatment for tracheobronchial stenoses/occlusions involving in carina. The aim of this study is to investigate the feasibility and efficiency of argon plasma coagulation (APC) and bifurcated covered Z-type stents (CZTS) placement for the treatment of tracheobronchus stenoses and esophagorespiratory fistula (ERF).

**Methods:**

Thirty-two cases with airway disorders were retrospectively reviewed for the treatment of APC and CZTS placement. Under the bronchoscopic guidance, APC was first given in patients with airway stenoses, then CZTS was placed under the fluoroscopic and flexible bronchoscopic guidance either with airway stenoses or ERF.

**Results:**

APC was first given in 19 patients with airway stenoses. Airway structures were significantly improved after APC procedure (57.4%-72.1% before APC *vs* 12.8%-25.8% after APC). Thirty-two CZTS were technically successfully placed in 30 out of 32 cases involving in carinal bifurcation. After APC and stent placement, short breath index was significantly reduced and karnofsky physical score (KPS) increased obviously. The endobronchial defects were successfully palliated with bifurcated CZTS placed under fluoroscopy and flexible bronchoscopy in 12 out of 13 patients with ERFs. There is no bleeding introprocedure. Mucous retention was very common in all types of the stents less than 2 weeks after stenting, which required debridement by bronchoscopy and could be prevented by normal saline solution nebulizations. Granuloma developed at the ends of stents after 1 month of stenting which all of them were treated successfully with APC and CO_2_ cryosurgery.

**Conclusion:**

Utilization of APC and retrieval bifurcated CZTS placement is a simple and safe protocol for the management of airway complex stenoses involving the tracheal carina and ERF.

气管下端、隆突和双侧主支气管发生的狭窄称为多发性狭窄或复合狭窄，常由晚期食管癌和肺癌引起，可分为管壁型、管内型、管外型(周围转移淋巴结或肿瘤压迫)病变，尚不能以单枚内支架有效地解除全部气道狭窄。单纯用氩等离子体凝固(argon plasma coagulation, APC)能快速清除阻塞气道内的病变，但术后易复发。国内外已有作者^[1-3]^使用不同类型的倒Y型一体化内支架治疗气道复合狭窄，临床取得满意疗效。近年来作者采用国产分叉型Sigma被膜金属支架(covered Z-type stents, CZTS)置入联合APC治疗气道复合狭窄和气管食管瘘，获得一定临床经验，现报告如下。

## 1 资料与方法

### 1.1 临床资料

回顾性分析我院2006年1月-2009年12月的32例放置分叉型支架患者，男25例、女7例，年龄46岁-82岁。良性病变14例、恶性病变18例。气管下段30例(其中合并气管上端1例)、隆突18例，右主支气管16例，左主支气管15例，其中同时合并多处病变25例。同时伴有气管下端气管食管瘘14例(其中5例为置入食道支架后支架上端机械损伤所致)，气管上端气管食管瘘2例(均为食道支架损伤所致)，支气管食管瘘1例(放疗所致)。管内型病变15例，管壁型病变29例，管外压迫型病变13例，其中28例为混合型病变(同时伴有两种以上病变)。

### 1.2 治疗方法

#### 1.2.1 氩等离子体凝固

电子支气管镜为PENTAX EPM-3500型主机(EB-1530T3和EB-1830T3)，APC为德国产CESEL 3000型。

19例管内型或管壁型患者采用电子支气管镜引导下的APC。按常规气管镜检查进行准备和操作。所有患者均采取改良神经安定镇痛术联合局部麻醉的方法进行，不需机械通气^[4]^。将APC探针通过气管镜活检孔伸出气管镜插入端(能见到探针标志为准)，在距病灶0.5 cm以内时开始烧灼。APC输出功率为30 W-50 W，氩气流量为0.8 L/min-1.6 L/min，每次烧灼深度不超过3 mm。并不断用活检钳或CO_2_冷冻取出凝固的组织。术中用心电监护仪严密监护心电、血压、氧饱和度和呼吸的变化。

#### 1.2.2 支架置入方法

被膜Z型支架(CZTS)由江苏西格玛医用实业有限公司生产，注册证号为国食药监械(准)字第2007第3461359号。骨架结构为Z型不锈钢，被膜为高强度医用硅橡胶薄膜^[4]^。骨架丝径0.4 mm-0.5 mm，支架直径10 mm-24 mm，长度12 mm-100 mm。根据需要，可制成直筒型支架、L型支架和Y型气管分叉支架。支架及其分支的长度和直径可根据临床需要定制。一般支架气管部直径16 mm-18 mm，长度30 mm-90 mm；左主支气管部直径10 mm-13 mm，长度15 mm-40 mm；右主支气管部直径10 mm-14 mm，长度10 mm-15 mm。

Y型及L型支架置入：根据胸部纵隔窗和气管镜观测气道狭窄位置、长度、程度以及引起管腔狭窄的原因，测量正常气管和主支气管的直径，测量左右主支气管长度(即分支开口与隆突距离)。根据测量结果选择CZTS的规格。一般支架长度大于狭窄段1 mm-2 cm，支架左右分支部短于左右主支气管长度，避免遮盖左右主支气管以远分支。气管支架口径等于正常段气管平均口径[(矢状径+横径) /2]。封闭气管瘘时气管支架口径等于正常段气管矢状径。

已行APC治疗的患者需在APC术后1周再行支气管镜清理坏死物后，再择机置入气道支架。Y型及L型支架需在支气管镜和X线透视双重引导下置入。先在气管镜引导下将导丝放入左侧支气管，再将带导引头的气管支架输送鞘在X线透视下送入左侧支气管，立即撤去固定插销，抽出输送鞘内芯，保留鞘管维持呼吸通畅，将已装有支架的内管送进鞘管内，注意内管把手上的定位孔方向。在X线监视下释放支架。支架定位适中后，固定内管后方的顶推管，后退鞘管，先将分叉的长臂释放在左支气管内，短臂释放在气管内，然后下推支架，短臂则自动进入右支气管内。放置支架后，抽出顶推管及内管，同时用气管镜观察支架的释放情况，并观察患者呼吸困难是否缓解，如支架位置正确而患者呼吸困难并未缓解则要分析原因，必要时取出支架。如支架位置偏低，可提拉鞘管上方的调整尼龙线，使支架上移，定位准确后剪断并抽出尼龙线、退出鞘管即可。如支架位置不当，应将支架拉出体外后重新放置。

L型内支架放置方法：定位同上，释放方法同Y型支架，支架的缺口需对准对侧支气管口，达预定部位后支架直接释放即可。

直筒型(I型)支架单独在支气管镜引导下放置即可。

支架上提或回收方法：在局部麻醉下将支气管镜插到气管内支架上方，将特制支架回收器沿活检孔插入，在支架上方将回收钩推出套管，用小钩沿着支架内壁上方向上滑动，钩到支架上口内的深色回收线，将塑料套管下推压住小钩，固定回收线。将回收套管上提，可见支架上端回缩，上提支架到合适的位置或直接拉出体外。

#### 1.2.3 随访

APC术后1天-3天再次行支气管检查，清除坏死物质，必要时再次APC烧灼。支架置入后3天-7天拍摄胸部X线片及气管镜检查，以了解支架的位置及肺膨胀情况。每隔1个月-2个月，经电话或门诊随访，了解患者的病情，重点了解患者咳嗽、呼吸困难、咯血及疼痛、出血等并发症情况。

## 2 结果

### 2.1 APC治疗结果

由表 1可见，术后气道阻塞较术前明显减轻。行APC治疗的19例患者气促指数术前为3.6±0.8，术后为1.6±0.1(*P*＜0.01)；KPS评分术前为29.4±1.9，术后为53.9±3.5(*t*=6.15, *P*＜0.01)。其中有2例完全性右全肺不张的患者经APC处理后堵塞管腔的肿瘤基本清除，肺全部复张。

**1:** APC治疗前后气道阻塞程度 Airway obstruction (OBS) before and after argon plasma coagulation (APC)

Location	*n*	OBS before APC (%)	OBS after APC(%)	*P*
Trachea	15	57.4±3.9	12.8±2.4	＜0.001
Left bronchus	13	67.3±6.5	25.8±1.9	＜0.001
Right bronchus	12	72.1±8.1	25.4±4.8	＜0.001

### 2.2 支架置入情况

30例技术上成功放置32个支架，其中28例均1次成功，另有4例第1次失败(3例Y型支架，因隆突较宽，未能置入。2例均改用L型+I型支架获得成功，1例改用I型支架置入后又咯出，未再放置。1例L型支架，因右肺癌行右全肺切除，术后放疗出现气管塌陷，置入L型支架出现支架膨胀不全致患者窒息，随即将支架取出，给予插管行机械通气治疗，复苏成功)。其余患者术中、术后未出现窒息、大出血、气管破裂等并发症。30例成功放置支架的患者气促指数术前为3.6±0.2，术后为1.5±0.2(*t*=7.42, *P*＜0.001)；KPS评分术前为45.5±2.9，术后为70.3±3.2(*t*=5.74, *P*＜0.001)。13例气管下端食管瘘的患者12例临床痊愈(其中1例食管淋巴瘤术中损伤致气管食管瘘的患者1年后将支架取出，瘘口完全愈合)，患者的咳嗽、咯痰症状明显缓解，能正常进食水；1例食管癌术后发生食管气管瘘和食管纵隔瘘，在食道和气管内分别置入食道支架和Y型支架，2个瘘口均有效封闭(图 1)；1例为气管下段和两侧支气管3 cm巨大瘘口，Y型支架难以有效封堵，1周后死于全身衰竭；2例堵瘘效果不佳(均为气管上段瘘，1例Y型支架未能封住瘘口，再置入I型支架，也未能奏效；1例置入I型支架也未能奏效)，患者的咳嗽、咯痰症状虽有好转，但仍不能进食水，其中1例因疼痛较重(与食道支架抵抗)，1周后将Y型支架取出(表 2)。

**1:**
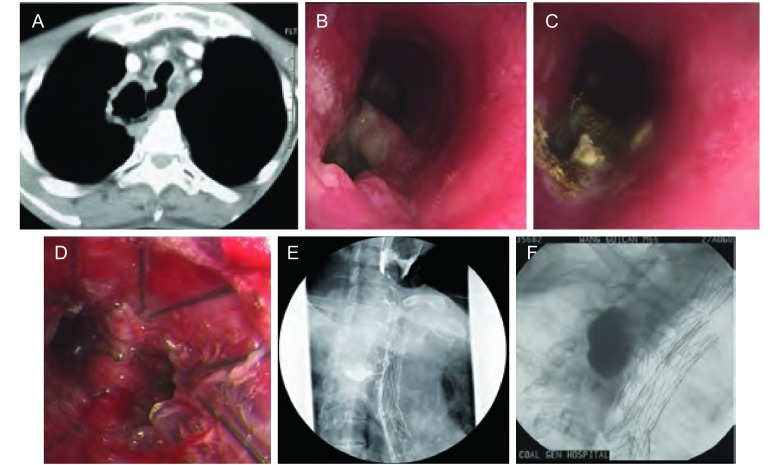
食管癌术后合并食管气管瘘和食管纵隔瘘的综合治疗。A：CT可见气管与食管相通；B：气管镜可见气管下端膜部2 cm×1.5 cm瘘口；C：用APC将瘘口周围的肿瘤烧灼；D：置入Y型气管支架将瘘口封闭；E：食管碘油造影显示气管食管瘘口封闭，但造影剂流到纵隔内；F：放置食管支架，将食管纵隔瘘口封闭。 Multiple modalities for the treatment of ERF and esophagomediastinal fistula in a patient with esophagus carcinoma postesophagostomy. A: CT showed that there is a channel between esophagus and trachea; B: Bronchoscopy showed that a fistula (2 cm×1.5 cm) is located in the back of trachea; C: Tumor around trachea fistula was ablated by APC; D: Y-shaped covered stent was inserted to seal the fistula; E: Esophageal hysterosalpingography showed that esophageal-trachea fistula was completely sealed, but contrast agent was drainage to mediastinal which suggested that esophageal-mediastinal fistula existed; F: Esophagus stent was inserted to seal the esophageal-mediastinal fistula.

**2:** 疾病及置入支架类型 Diseases and types of stent

Diseases	*n*	Types of stent
Benign		
Recurrent polychondritis	1	Y1
GT posttracheotomy	1	L1+Y1
Burn of trachea	1	Right-arm L1+Left L1
ERF after esophagus surgery	1	Y1
ERF after esophagus stent	6	Y6^*^
Tracheal collapse after radiotherapy	2	Y1, L1
ERF after radiotherapy	2	Y1, Right-arm L1+Left L1
Malignant		
Cystic adenocarcinoma	3 (ERF 1)	Y3
Squamous cell carcinoma	6 (ERF 2)	Left-arm L1 Right-arm L2 Y3
Mixed sqaumous-adenocarcinoma	1	Right-arm L1
Adenocarcinoma	2	Y2
Small cell lung cancer(SCLC)	1	Y1
Metastasis of esophagus carcinoma	4 (ERF 2)	Y4, L1
Metastasis of thyroid carcinoma	1	Y1
Total	32	34
GT: granulation tissue; ERF: esophagorespiratory fistula; Y5: 5 Y-shaped stents; Right-arm L: Right-arm L-shaped stent; Left-arm L: Left-arm L-shaped stent.		

所有支架置入后1周左右，有较多粘稠分泌物粘附，需多次用气管镜将分泌物清除，并配合每日两次生理盐水雾化吸入。特别是气管食管瘘的患者，分泌物较多，需及时清理。一般1个月左右分泌物会明显减少。

支架置入后2天-3天，支架两端的气管黏膜表面有白膜覆着，1周左右可形成一层坏死物质粘附，患者咳嗽较重，良性气道狭窄的患者更为明显，需用气管镜将坏死物清除。本组7例良性气道狭窄置入支架后1个月左右均可见支架两端有肉芽肿形成，可用APC及冷冻将肉芽肿清除。其中1例气管切开的患者，置入支架后气道发生弥漫性肉芽肿样改变，在支架的两端很快会形成堵塞性肉芽肿，需每月用支气管镜清理。1例复发性多发性软骨炎的患者置入支架3年后方因肉芽肿再狭窄而行气管镜清理。

19例气道狭窄先行APC的患者，4例在APC后同时置入支架，结果有大量的坏死物潴留，特别是隐藏在支架后方的部分难以清除，支架两端也有大量的坏死物，极易造成管腔堵塞。其余15例支架均在APC 1周后进行，先将分泌物清除，置入支架后分泌物明显减少，但恶性肿瘤的患者分泌物仍多于良性病变，肉芽肿形成时间要长于良性病变，程度亦较轻，结合APC和冷冻可有效防治肉芽肿的形成。

## 3 讨论

对于不能手术切除的管外型气道病变，内支架置入是最佳治疗方案。但对不能手术的管壁型和管内型气道病变，传统上仍通过置入内支架的方法解除气道梗阻^[5]^。但近年来随着介入支气管镜学的发展，消融治疗(冻切或热烧灼)成为其首要治疗方法。由于内支架置入后肿瘤负荷未减轻，术后仍易复发，所以，置入支架前应先减瘤负荷再置入支架预防其梗阻是可行的序贯治疗方法^[6]^。

目前应用较为广泛的热消融方法为APC ^[5, 6]^。其工作原理是利用高频电刀提供的高频、高压电流，将氩气电离成氩等离子体，这种氩气离子具有极好的导电性，可连续传递电流，减少损伤组织的氧化、炭化(冒烟、焦痂)，所以在切割时冒烟少，组织烫伤、坏死层浅。APC可使组织得到表浅而均匀一致的凝固(最深不超过3 mm)，因而可逐层切除面积较大的狭窄组织(瘢痕或肿瘤)，又可快速止血，成为消除器质性气道狭窄的有效手段。本组19例有气道狭窄的患者进行了APC治疗，术后患者的气促指数和KPS评分均较术前有明显改善。特别是有2例完全性右全肺不张的患者经APC处理后右肺基本复张，这是支架所难以做到的。APC还可烧灼气管食管瘘瘘口的肿瘤，清除部分瘤体，也利于止血，但烧灼范围不宜太大，否则会扩大瘘口。

APC术后如不采取其它有效措施，管腔还会发生狭窄。置入单管状内支架仅解除一侧主支气管狭窄而牺牲另一侧肺，或在气管和双侧主支气管分别置入两枚单分支状内支架，或在气管和双侧主支气管分别置入三个单管状支架才能完全解除气道的复合性狭窄，这样不仅增加了患者的费用和痛苦，而且技术要求高、操作困难，增加了手术并发症的发生率。

对于气管下段、隆突区和双侧主支气管多处病变造成的狭窄，只有置入倒Y型一体化内支架才能够完全解除隆突区的复合性气道狭窄，克服了多次置入操作，解除气道狭窄更符合解剖学和生理学要求^[3]^。本研究采用一种新型的分叉型CZTS支架，取得了良好的治疗效果。

CZTS分叉型内支架根据需要设计成Y型或L型，置入方法比管状内支架操作相对复杂，手术操作者应熟练掌握该支架释放技术，防止支架置入过程中患者窒息。一般先行支气管镜检查，清除气道内的肿瘤和分泌物，由于支架置入前已行APC等治疗，气道内的梗阻已大部分解除，结合神经安定镇痛术，所以置入支架时患者无多大痛苦。在支气管镜直视下插入导丝，又在导丝和X线引导下插入呼吸性鞘管，保证了患者通气，大大减少了窒息的风险。Y型支架释放过程中应注意先将左侧支架退出，再顺势将右侧支架推进到右侧支气管内。支架释放完毕后再行支气管镜检查和X线透视，以确认支架释放准确、无误。如果隆突较宽，则可结合L型和I型支架，分别置入到两侧支气管内，本组2例均获成功。本组1例L型支架术中出现窒息，主要是患者右全肺切除，术后又给予放疗，气道瘢痕狭窄，支架置入后难以完全释放。因此，术前应充分估计病情，必要时在全麻、硬质镜下放置较为安全。

支架置入后，解除了大气道堵塞，呼吸困难得以改善，特别是有气管食管瘘的患者，由于有效地封堵了瘘口，同时支架的两侧支分别卡在两侧支气管内，也能有效预防分泌物流到支气管内，患者咳嗽、咯痰等症状明显缓解，肺部感染很快得以控制，并且可以进食，提高了患者生活质量，延长患者生命。值得注意的是，本组有5例食管癌患者置入网状食管支架后，蘑菇头状的支架上端因机械损伤导致气管食管瘘，先将食道支架取出后，再放置Y型气管支架，瘘口均有效封堵，患者均能正常进食水，毋须再在食道内放置支架。同时应注意，食道支架置入后如果患者疼痛难忍，并出现饮水呛咳，应及时查找瘘口，尽早将食道支架取出，放置分叉型气道支架。一般气管中下段的瘘口放置分叉型支架后可取得良好效果，而发生于气管上段的瘘口则需同时结合食道支架置入。

支架置入后1周内分泌物较多，需及时用气管镜清理，并配合雾化吸入。一般1个月左右分泌物会明显减少。

支架置入后还需注意肉芽肿的问题。置入支架后1月左右均可见支架两端有肉芽肿形成，良性气道狭窄者发生肉芽肿比恶性狭窄者时间短，程度重，需用APC将肉芽肿清除，再结合冷冻以防肉芽再生。

总之，APC结合分叉型CZTS置入治疗气道复合狭窄，操作技术安全、解除狭窄立竿见影，疗效可靠，值得临床进一步推广应用。

## References

[1] Han XW, Wu G, Gao XM (2007). The technique study and primary clinical application of inverted Y-shaped self-expandable metal airway stent. J Interv Radiol.

[2] Dutau H, Toutblanc B, Lamb C (2004). Use of the Dumon Y-stent in the management of malignant disease involving the carina: a retrospective review of 86 patients. Chest.

[3] Wang PW, Wu G, Han XW (2009). The treatment of airway complex stenoses with inverted Y-shaped self-expandable metal stent. Current Med.

[b4] 4Wang HW ed. Clinical practice of electronic bronchoscopy. 1st ed. Beijing: China Med Sci & Tech Press, 2009. 67. 王洪武主编. 电子支气管镜的临床应用. 第1版. 北京: 中国医药科技出版社, 2009. 67.

[5] Morice RC, Ece T, Ece F (2001). Endobronchial argon plasmacoagulation for treatment of hemoptysis and neoplastic airwayobstruction. Chest.

[6] Wang HW (2008). Endobronchial argon plasma coagulation for the treatment of airway diseases. J Clin Rehabil Tis Engine Res.

